# Influence of root canal instrumentation and obturation techniques on intra-operative pain during endodontic therapy

**DOI:** 10.4317/medoral.18234

**Published:** 2012-05-01

**Authors:** Jenifer Martín-González, Marta Echevarría-Pérez, Benito Sánchez-Domínguez, Maria L. Tarilonte-Delgado, Lizett Castellanos-Cosano, Francisco J. López-Frías, Juan J. Segura-Egea

**Affiliations:** 1 DDS, Doctoral fellow, Department of Endodontics, School of Dentistry, University of Sevilla, Sevilla, Spain; 2 MD, PhD, DDS, Assistant Professor, Department of Endodontics, School of Dentistry, University of Sevilla, Sevilla, Spain; 3 MD, PhD, DDS, Professor, Department of Endodontics, School of Dentistry, University of Sevilla, Sevilla, Spain

## Abstract

Objective: To analyse the influence of root canal instrumentation and obturation techniques on intra-operative pain experienced by patients during endodontic therapy. Method and Materials: A descriptive cross-sectional study was carried out in Ponferrada and Sevilla, Spain, including 80 patients (46 men and 34 women), with ages ranged from 10 to 74 years, randomly recruited. Patient gender and age, affected tooth, pulpal diagnosis, periapical status, previous NSAID or antibiotic (AB) treatment, and root canal instrumentation and obturation techniques were recorded. After root canal treatment (RCT), patients completed a 10-cm visual analogue scale (VAS) that ranked the level of pain. Results were analysed statistically using the Chi-square and ANOVA tests and logistic regression analysis. Results: The mean pain level during root canal treatment was 2.9 ± 3.0 (median = 2) in a VAS between 0 and 10. Forty percent of patients experienced no pain. Gender, age, arch, previous NSAIDs or AB treatment and anaesthetic type did not influence significantly the pain level (p > 0.05). Pain during root canal treatment was significantly greater in molar teeth (OR = 10.1; 95% C.I. = 1.6 - 63.5; p = 0.013). Root canal instrumentation and obturation techniques did not affect significantly patient’s pain during root canal treatment (p > 0.05). Conclusion: Patients feel more pain when RCT is carried out on molar teeth. The root canal instrumentation and obturation techniques do not affect significantly the patients’ pain during RCT.

** Key words:**Anaesthesia, endodontic pain, pulpitis, root canal instrumentation, root canal obturation, rotary files.

## Introduction

Root canal therapy is one of the most common procedures (1), as well as one of the most feared dental procedures (2). Therefore, managing pain is a challenge in the clinical practice of endodontics, and the main aspect by which the skill of the clinician is often judged (2). However, managing the pain and distress of patients can be frustrating, especially when the root canal treatment (RCT) itself appears to initiate its onset. Indeed, the result can be distressing to both the patient and the operator (3). In contrast, the elimination of pain enhances the confidence of patients.

Most studies concerning endodontic pain have investigated the patient’s pain experienced after the root canal treatment, i.e. the post-operative pain (4-6). Mechanical factors, including root canal instrumentation techniques (6-8), over instrumentation or extrusion of root-filling materials (9), have been associated to the presence of postoperative pain. However, endodontic patients usually associate fear of pain with the procedure itself, not with the post-treatment period (10). Mean intra-operative pain levels ranging 0.8 to 2.3 in a visual analogical scale (VAS) have been reported during RCT (11-13). Several factors have been analysed in relation with endodontic intra-operative pain. Age, tooth type and length of the treatment were factors associated with increased risk for pain experienced during the procedure (12,13). RCT in teeth with irreversible pulpitis and acute apical perio-dontitis has been reported to be more painful, as well as interventions longer than 45 min (13).

Rotary root canal instruments manufactured from nickel-titanium alloy as well as continuous wave of compaction have proved to be a valuable adjunct for root canal therapy. However, no data is available on the effect of root canal instrumentation and obturation techniques, on intra-operative pain during RCT. The aim of this study was to analyse the influence of root canal instrumentation and obturation techniques on intra-operative pain experienced by patients during endodontic therapy.

## Material and Methods

Subjects

Eighty patients (46 men and 34 women), with ages ranging from 10 to 74 yr (mean: 40.2 ± 16.5 yr; median: 40), were questioned after undergoing root canal treatment in relation to their pain perception. Patients were randomly recruited in two private dental clinics one in Ponferrada (León, Spain) and other in Seville (Seville, Spain). The experiments were undertaken with the understanding and written consent of each subject and have been conducted in full accordance with ethical principles, including the World Medical Association Declaration of Helsinki. The study has been independently reviewed and approved by the Ethic Committee of the Dental Faculty of the University of Sevilla, Sevilla, Spain.

Prior to treatment, a thorough clinical and radiological examination was carried out. Patient gender and age, affected tooth, pulpal diagnosis (normal, irreversible pulpitis or necrotic), periapical status (normal, acute apical periodontitis and chronic apical periodontitis), and previous NSAID or antibiotic (AB) treatment were recorded ( Table 1).

**Table 1:**
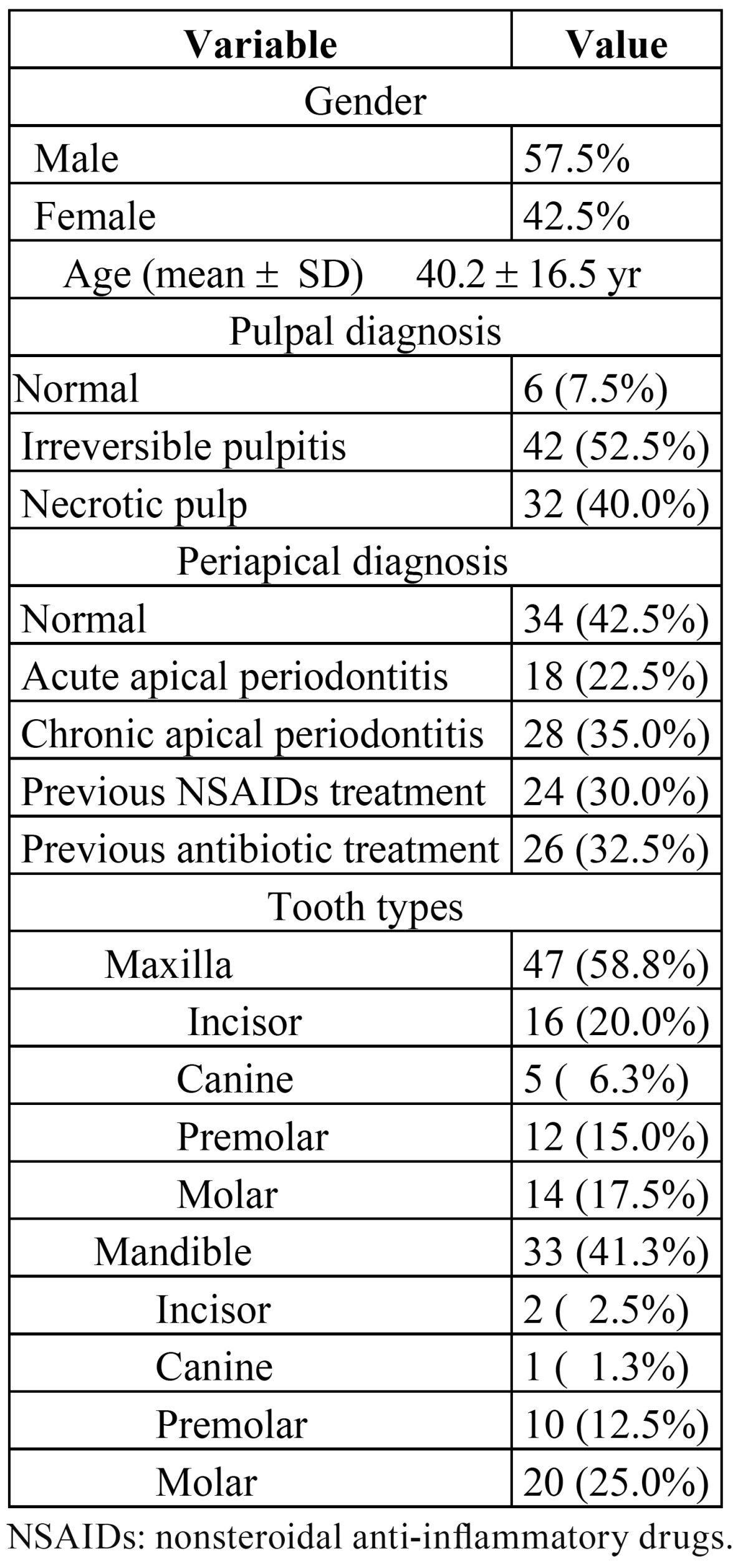
Variables recorded prior to root canal treatment and distribution by tooth type and arch of the root-filled teeth.

Root canal instrumentation techniques

The tooth was anaesthetized with Articaine 4% with 1:100,000 Epinephrine or, in hypertensive patients (20.0%), Mepivacaine 3% without vasoconstrictor, the volume of anaesthetic and type of injection being at the discretion of the dentist. Following an adequate anaesthesia and isolation with rubber dam, an endodontic access cavity was established. After apical patency, the working length was estimated using an apex locator (Dentaport ZX, Morita, Tokyo, Japan) and then confirmed with a periapical radiograph.

Root canals were cleaned and shaped using either step-back technique, with hand instrumentation (n = 22), or crown-down technique with rotary instrumentation (n = 58).

1) Step-back technique with hand instrumentation:

The root canal treatment was carried out involving canal shaping with hand files using the step-back technique and saline irrigation. Coronal flaring was carried out with Gates Glidden burs (sizes # 3 and 4) (Dents-ply Maillefer, Ballaigues, Switzerland). The canal was cleaned and shaped by hand with K-Flexofiles (Dents-ply Maillefer, Ballaigues, Switzerland) under irrigation with 5.25% sodium hypochlorite (NaOCl) and 17% EDTA.

2) Crown-down technique with rotary instrumentation:

Canals were prepared using ProTaper rotary instruments (Dentsply-Maillefer, Ballaigues, Switzerland) in low-torque motors with torque control and constant speed of 300 r.p.m., using 5.25% sodium hypochlorite and 17% EDTA as irrigants. Canals were enlarged with S1 and S2 files, which were used in a gentle pumping and brushing action as recommended by the manufacturer. Then, apical instrument F1 (D0=0.20 mm/taper 7% at the first mm), F2 (D0=0.25mm/taper 8% at the first mm) or F3 (D0=0.30 mm/taper 9% at the first mm) were employed.

Root canal obturation techniques

After cleaning and shaping, canals were dried and obturated. Root canals instrumented using only hand files and step-back technique (n = 22) were obturated by cold lateral compaction of gutta-percha (Dentsply Maillefer, Ballaigues, Switzerland) and sealer (AH Plus, Dentsply DeTrey, Konstanz, Germany). Some root canals prepared with rotary files were also obturated by cold lateral compaction (n = 22) and the others (n = 36) were obturated with AH Plus and gutta-percha using the continuous wave of compaction technique (System B, EIE Analytic Technology, Redmond, WA, USA). Treatment was completed during the same appointment and, immediately, a periapical radiograph was taken. Working length, root canal instrumentation technique, maximal apical file, obturation technique and length of root filling for each treated canal/tooth were recorded. Root filling length was radiographically evaluated as adequate if ending ≤ 2 mm from, or flush with, the radiographic apex.

Pain assessment

Immediately, each patient received instruction on how to use a 10-cm visual analogue scale (VAS) (14) to assess pain. As soon as each patient self-recorded his/her pain by ranking the level of pain experienced during treatment, he/she was informed verbally about the aim of the study. Then, this score was converted to a numerical value between 0 and 10 and to a verbal scale (none, slight, moderate, intense, and unbearable).

Statistical analysis

Raw data were entered into Excel® (Microsoft Corporation, Redmond, WA, USA). Frequency distributions and contingency table analyses were used to describe and compare independent variables with patient-reported pain (Chi-square test and ANOVA, significance level α = 0.05). Experienced pain variables were analysed first as continuous variables and then were dichotomized into high or low categories according to the sample distribution and previous literature reports on VAS (13,15). Statistical logistic regression modelling technique was used.

## Results

The mean pain level during root canal treatment was 2.9 ± 3.0 in a VAS scale between 0 and 10 (median = 2). Pain was absent in 40% of the cases. The pain experienced was slight, moderate and intense in 25%, 27.5% and 7.5% of the cases, respectively. No intervention resulted in unbearable pain. Mean pain levels did not differ between men (3.2 ± 3.9) and women (2.6 ± 3.0) (p > 0.05). Thirty-five percent of men and forty-seven percent of women did not experience pain during the treatment (p > 0.05).

Univariate logistic regressions were run with age, gender, tooth type, arch, pulp vitality, irreversible pulpitis, acute apical periodontitis, previous NSAIDs, previous AB treatment and anaesthetic type ( Table 2). Age, gender, arch, pulp vitality, acute apical periodontitis, previous NSAIDs or AB treatment and anaesthetic type did not influence significantly the pain level (p > 0.05). However, root canal treatment was significantly more painful in molar teeth (OR = 9.8; 95% C.I. = 1.8 – 53.1; p < 0.01) and in teeth with irreversible pulpitis (OR = 4.4; 95% C.I. = 1.1 – 17.1; p < 0.05).

**Table 2:**
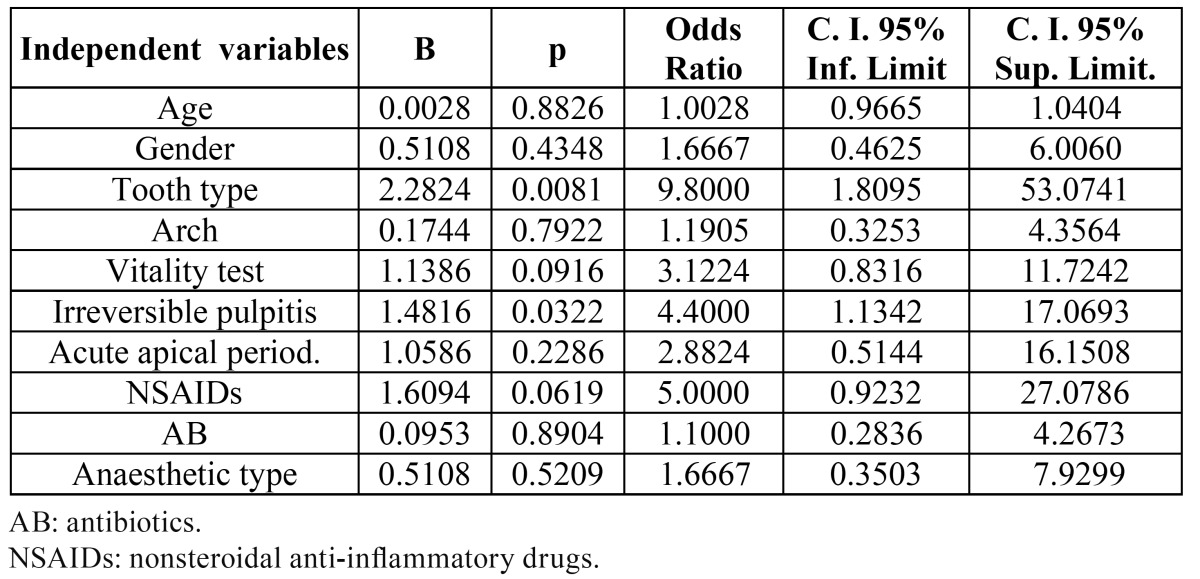
Univariate logistic regression analyse of the influence of the independent variables age, gender (male / female), tooth type (1 = molar / 0 = other), arch (1 = mandible / 0 = maxilla), vitality test (1 = positive / 0 = negative), pulpitis (1 = present / 0 = absent), acute apical periodontitis (1 = present / 0 = absent), previous NSAIDs (1 = present / 0 = absent), previous AB (1 = present / 0 = absent), anaesthetic type (1 = articaine, 0 = mepivacaine) on the dependent variable “pain experienced during root canal treatment” (1 = absent / 0 = present).

ANOVA analysis showed significant differences in pain perception during root canal treatment in relation to the root canal instrumentation and obturation techniques (p < 0.02) ( Table 3). Root canal instrumentation using step-back technique with hand files produced significantly more pain perception than rotary files (p = 0.003). Lateral compaction (LC) produced significant more pain perception than continuous wave of compaction (CWC) technique (p = 0.004). Patients whose root canals were instrumented with hand files and step-back technique and obturated by cold lateral compaction of gutta-percha and sealer (SB-LC) reported pain in 36.4% of cases, as well as patients whose canals were prepared using rotary files and were obturated by cold lateral compaction. Patients whose canals were prepared using rotary files but were filled using the conti-nuous wave of compaction technique (RF-CWC) reported less pain (p < 0.05).

**Table 3:**
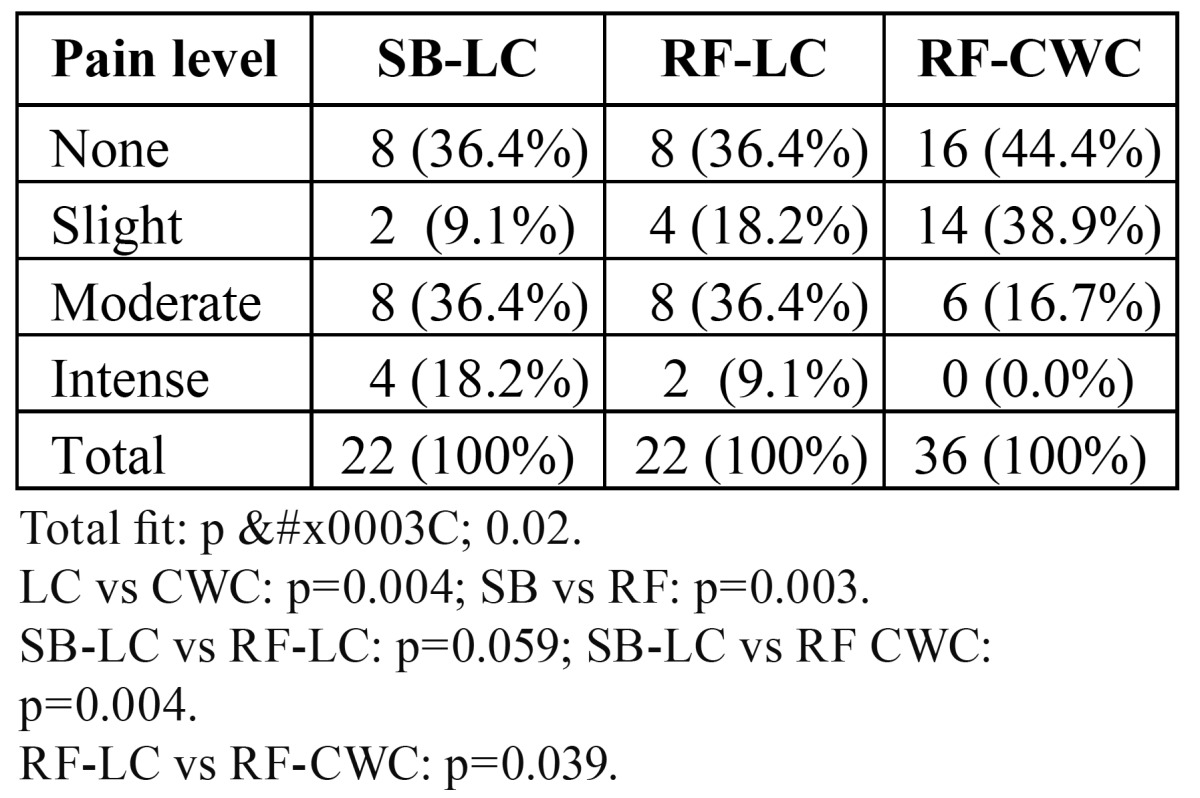
Pain experienced during endodontic therapy by root canal instrumentation and obturation techniques. SB-LC: step-back with hand files and lateral compaction; RF-LC: rotary files and lateral compaction; RF-CWC: rotary files and continuous wave of compaction.

When multivariate logistic regression analysis was run ( Table 4) with tooth type, pulp vitality, irreversible pulpitis, acute apical periodontitis, previous NSAIDs, root canal instrumentation technique and root canal obturation technique as covariates, only tooth type (molar tooth = 1) (OR = 10.1; 95% C.I. = 1.6 - 63.5; p = 0.013) remained significantly associated with increased risk for pain experienced during the procedure. Root canal instrumentation and obturation techniques did not influence significantly the pain level (p > 0.05).

**Table 4:**
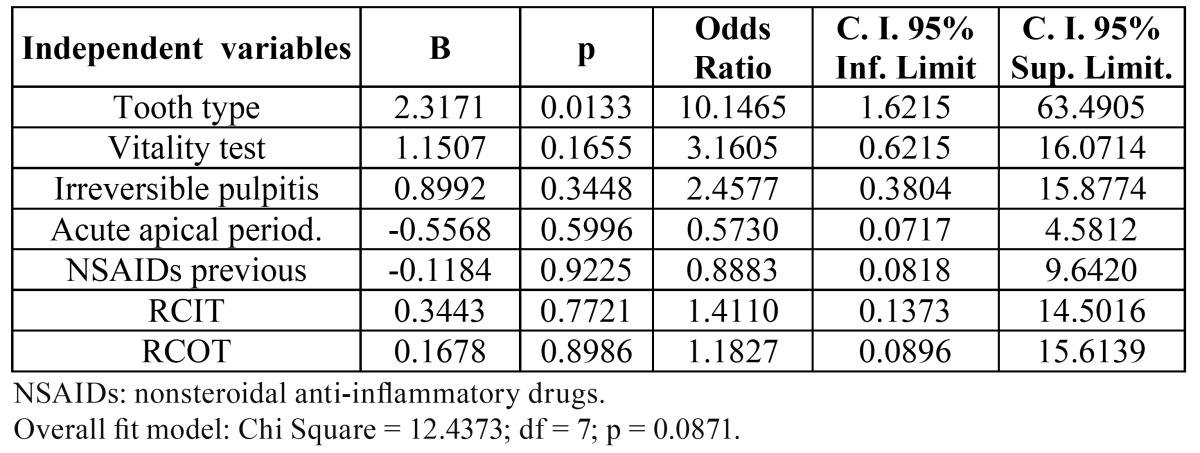
Multivariate logistic regression analyse of the influence of the independent variables tooth type (1 = molar / 0 = other), vitality test (1 = positive / 0 = negative), pulpitis (1 = present / 0 = absent), acute apical periodontitis (1 = present / 0 = absent), previous NSAIDs (1 = present / 0 = absent), root canal instrumentation technique (RCIT) (1 = hand files / 0 = rotary files) and root canal obturation technique (RCOT) (1 = lateral compaction / 0 = continuous wave of compaction) on the dependent variable “pain experienced during root canal treatment” (1 = absent / 0 = present).

## Discussion

Dental patients have become increasingly less tolerant of any dentist or dental procedure that causes pain. In endodontics, offering adequate local anaesthesia is essential for successful patient management and represents a practice-building strategy that increases both patient loyalty and treatment acceptance.

Endodontic pain management must encompass all aspects of treatment: preoperative pain control includes accurate diagnosis and anxiety reduction; intraoperative pain control revolves around effective local anaesthetic and operative techniques; and postoperative pain management can involve a variety of pharmacologic agents (2). However, few studies analyse the pain experienced during root canal treatment (11-13). Furthermore, as long as we know, no studies are available on the effect of root canal instrumentation and obturation techniques on the pain experienced by patients during endodontic therapy.

In this study the influence of the type of root canal instrumentation and obturation techniques on the level of patient pain experienced during root canal treatment have been analysed. The results revealed that forty percent of patients did not feel pain during root canal treatment, but about 35% of patients experienced moderate-to-intense pain. Assessment of the experienced intraoperative pain was carried out using a visual analogue scale (VAS), a valid and reliable method widely used in the endodontic literature (3,6,13,16,17). Patients were told the aim of the study after self-recorded their pain. Thus, the so-called Hawthorne effect (18), i. e. the mere awareness of participants in an investigation can alter the way in which a person behaves, was minimized.

The mean pain level during root canal treatment found in the present report was 2.9 ± 3.0 (median = 2) in a VAS between 0 and 10. Intense pain only was experienced by 7.5% of patients. Previous investigations using VAS between 0 and 100 reported comparable results. Thus, in the study developed by Rousseau et al. (11) the mean pain experienced during root canal treatment was 7.7; Watkins et al. (12) reported the mean pain level during root canal treatment was 22.7 ± 19.9, meaning that 22.6% of patients felt high pain levels. Segura-Egea et al. (13) have reported a mean pain level of 1.2 ± 0.8 in a VAS between 0 and 10.

The mean levels of experienced pain did not differ by gender. Watkins et al. (12) reported analogous intraoperative pain levels in both sexes, although women anticipated higher pain levels than men. However, Segura-Egea et al. (13) reported a higher percentage of men (61%) who did not experience pain during treatment compared to women (47%) (p < 0.05). Gender differences in pain reports with women reporting more pain than men (19) and the reduction of pain thresholds in women (20) have been reported previously. Moreover, Polycarpou et al. (17) determined the prevalence of persistent den to-alveolar pain following nonsurgical and/or surgical endodontic treatment, concluding that female gender was an important risk factor associated with persistent pain after successful endodontic treatment. Khan et al. (21) found significantly higher levels of mechanical allodynia, defined as reduced mechanical pain thresholds, in women with irreversible pulpitis and acute periradicular periodontitis, compared to men.

The findings of the present study show that age did not correlated with pain levels. However, other studies have found that anticipated and experienced outcome levels significantly decreased with increasing age (12) as well as that patients older than 35 years felt less pain compared to patients aging 35 years and younger (13). There are no conclusive data that progressive loss of sensitivity to nociceptive stimuli occurs with age (22). Thus, the age-related decrease in pain is not thought to be attributable to changes in the physiological pain system.

Root canal treatment was significantly more painful in molar teeth (OR = 10.1; 95% C.I. = 1.6 - 63.5; p = 0.013) and correlated with the number of canals (OR = 3.3; 95% CI 1.4 – 7.6; p = 0.005). Previously, posterior teeth located in the mandibular arch have been reported to be associated significantly with higher levels of post-endodontic pain (23). Segura-Egea et al. (13) also reported significant differences in pain levels between treatments carried out in incisors and canines compared to premolars and molars. This difference may be related biologically to a greater number of canals and high frequency of bifurcated root canals in posterior teeth (12,24). The length of the treatment, longer in molar teeth, could also explain this result, taking into account the progressive decrease of the anaesthetic effect (25,26), together with the increase of the anxiety of the patient as the intervention extended. A previous study showed that the percentage of patients who did not feel pain decreased as the length of the procedure increased (13). However, other studies have not found differences in pain level in relation to tooth type (12).

Univariate logistic regression analysis demonstrated that root canal treatment in teeth with irreversible pulpitis was significantly more painful than that in teeth with normal or necrotic pulps (OR = 4.4; 95% CI 1.1 – 17.1; p = 0.03). Pain is a major complaint in irreversible pulpitis. Dummer et al. (27) found that 87% of patients who suffered from acute pulpitis reported severe pain, and that all patients who presented with AAP complained of severe pain. Owatz et al. (28) reported that the incidence of mechanical-allodynia in patients presenting with irreversible pulpitis was 57.2%, suggesting that periradicular mechanical-allodynia contributes to early stages of odontogenic pain because of inflammation of vital pulpal tissue. Thus, the reduced mechanical pain thresholds associated with mechanical-allodynia could explain that root canal treatment in teeth with irreversible pulpitis was significantly more painful than that in teeth with normal or necrotic pulps (13). Nevertheless, multivariate logistic regression suggested that only tooth type was a factor associated with increased risk for pain experienced during the procedure.

Univariate analysis showed significant differences in pain perception during root canal treatment in relation to the root canal instrumentation and obturation techniques (p < 0.02). Root canal instrumentation using step-back technique with hand files produced significantly more pain perception than rotary files (p = 0.003) and lateral compaction (LC) produced significant more pain perception than continuous wave of compaction (CWC) technique (p = 0.004). However, in the multivariate logistic regression model, neither the root canal instrumentation techniques nor the root canal obturation techniques remained associated with increased risk for intra-operative pain. No other studies are available comparing the effect of root canal instrumentation and obturation techniques on the experienced pain during endodontic therapy. Goreva & Petrikas (7), studying postobturation pain of different origin after endodontic treatment, reported that “crown down” preparation using completely rotating profile instruments and GT rotary files proved to be effective as regards prevention of postoperative pain. The effects of the technique used for root canal instrumentation on emergence of pain after endodontic therapy have been analyzed by Makeeva & Turkina (8). These authors compared sound tools of the Sonic system, ultrasound tools of the Satelec Suprasson system, full-wind tools of ProTaper and System GT as well as handy K-files. It was found that the least risk of pain emergence after endodontic treatment occurs with tooth canal widening by crown-down technique. Recently, Parirokh et al. (29) have studied the number of patients experiencing pain during endodontic therapy when penetrating dentin, when reaching the pulp chamber, and during canal instrumentation. Overall 60.5% of the patients felt pain, but instrumentation was less painful compared to access cavity. Iqbal et al. (30) investigated the incidence and factors related to endodontic flare-ups in nonsurgical root canal treatment and did not found differences in relation to root canal instrumentation and obturation techniques.

In conclusion, patients feel more pain when root canal treatment is carried out on molar teeth. The root canal instrumentation and obturation techniques do not affect significantly the patients’ pain during root canal treatment.
